# Omega-3 Fatty Acids Modulate Weibel-Palade Body Degranulation and Actin Cytoskeleton Rearrangement in PMA-Stimulated Human Umbilical Vein Endothelial Cells

**DOI:** 10.3390/md11114435

**Published:** 2013-11-08

**Authors:** Corinna S. Bürgin-Maunder, Peter R. Brooks, Fraser D. Russell

**Affiliations:** Inflammation and Healing Research Cluster, School of Health and Sport Sciences, University of the Sunshine Coast, Maroochydore, Queensland 4556, Australia; E-Mails: CBurgin@usc.edu.au (C.S.B.-M.); PBrooks@usc.edu.au (P.R.B.)

**Keywords:** omega-3 fatty acids, von willebrand factor, weibel-palade bodies, endothelial function, actin cytoskeleton

## Abstract

Long chain omega-3 polyunsaturated fatty acids (LC *n*-3 PUFAs) produce cardiovascular benefits by improving endothelial function. Endothelial cells store von Willebrand factor (vWF) in cytoplasmic Weibel-Palade bodies (WPBs). We examined whether LC *n*-3 PUFAs regulate WPB degranulation using cultured human umbilical vein endothelial cells (HUVECs). HUVECs were incubated with or without 75 or 120 µM docosahexaenoic acid or eicosapentaenoic acid for 5 days at 37 °C. WPB degranulation was stimulated using phorbol 12-myristate 13-acetate (PMA), and this was assessed by immunocytochemical staining for vWF. Actin reorganization was determined using phalloidin-TRITC staining. We found that PMA stimulated WPB degranulation, and that this was significantly reduced by prior incubation of cells with LC *n*-3 PUFAs. In these cells, WPBs had rounded rather than rod-shaped morphology and localized to the perinuclear region, suggesting interference with cytoskeletal remodeling that is necessary for complete WPB degranulation. In line with this, actin rearrangement was altered in cells containing perinuclear WPBs, where cells exhibited a thickened actin rim in the absence of prominent cytoplasmic stress fibers. These findings indicate that LC *n*-3 PUFAs provide some protection against WBP degranulation, and may contribute to an improved understanding of the anti-thrombotic effects previously attributed to LC *n*-3 PUFAs.

## 1. Introduction

Endothelial dysfunction, a corollary of hypertension, contributes to the development of inflammation and atherosclerosis [1]. Excessive exocytosis of endothelial storage granules containing pro-inflammatory and vasoconstrictor substances might therefore be implicated in the development of endothelial dysfunction. Weibel-Palade bodies (WPBs) are endothelial storage granules that release vasoactive substances such as von Willebrand factor (vWF), P-selectin and endothelin-1 [2]. The rod shape of WPBs is dependent on polymerisation of vWF and consequent tubular arrangement of mature vWF multimers within the granules [3]. Anchoring of WPBs to actin cytoskeleton via the small GTPase Rab27a/MyRIP complex prevents premature exocytosis and allows for full WPB maturation and assembly of high molecular weight vWF multimers [4,5]. Secretagogues include thrombin, histamine, fibrinogen and the protein kinase C (PKC) activator (and diacylglycerol analog), phorbol 12-myristate 13-acetate (PMA) [6,7,8,9]. Factors released by WPBs after endothelial activation contribute to inflammation associated with hypertension and thrombosis, where inhibition of exocytosis may attenuate this response [10,11,12].

WPB degranulation involves rearrangement of cytoskeletal actin and myosin microfilaments [13]. In particular, rearrangement of actin filaments into band-like stress fibers is associated with complete WPB degranulation, whereas remodeling of the cortical actin rim precedes degranulation of peripheral WPBs only [13]. It has further been shown that stimulation of human umbilical vein endothelial cells (HUVECs) with PMA results in longitudinal stress fiber formation as well as recruitment of actin filaments to WPBs undergoing exocytosis [14]. The consequent formation of a dynamic actin ring around the base of WPBs facilitates the release of vWF from the WPBs at the cell surface [14].

Increased dietary intake of oily fish, or supplements containing high levels of long chain omega-3 polyunsaturated fatty acids (LC *n*-3 PUFAs), reportedly improve cardiovascular health [15,16,17,18,19,20]. The cardiovascular benefits of LC *n*-3 PUFAs have been partly attributed to their incorporation into phospholipids of membrane lipid rafts [21]. Enrichment of lipid rafts with *n*-3 PUFAs can displace signaling proteins from the rafts resulting in suppression of T-cell activation [21,22]. It has also been shown that *n*-3 PUFAs can improve endothelial function [23,24], and decrease circulating levels of vWF [25,26]. However, the mechanisms for these effects are not fully understood. One possibility is that LC *n*-3 PUFAs attenuate the release of pre-stored substances from the endothelium to decrease circulating concentrations of pro-inflammatory mediators such as vWF. To test this hypothesis, we treated cultured HUVECs with LC *n*-3 PUFAs, docosahexaenoic acid (DHA) or eicosapentaenoic acid (EPA), and examined their ability to attenuate PMA-stimulated WPB degranulation as well as their effects on actin rearrangement.

## 2. Results and Discussion

### 2.1. PMA-Stimulated Degranulation of Weibel-Palade Bodies

**Figure 1:**
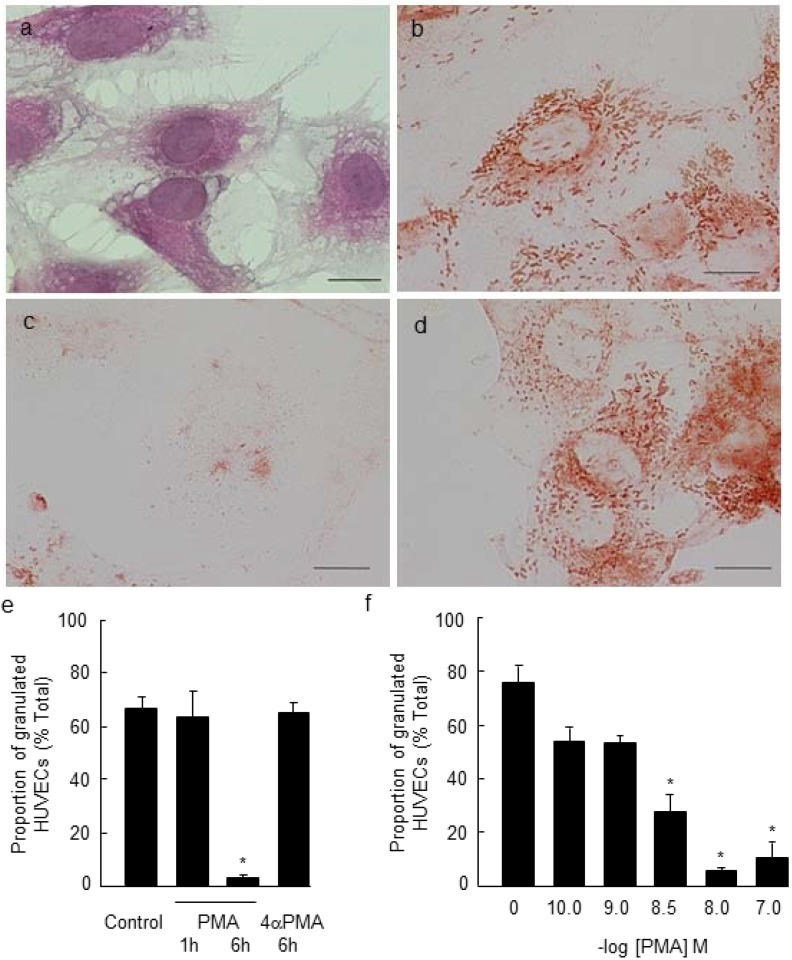
Effect of phorbol 12-myristate 13-acetate (PMA) and 4α-phorbol 12-myristate 13-acetate (4α-PMA) on Weibel-Palade body (WPB) degranulation. Human umbilical vein endothelial cells (HUVECs) were stained with hematoxylin and eosin to show cell morphology (**a**). WPBs within HUVECs stained positively for von Willebrand factor (**b**). Treatment of cells with 10 nM PMA for 6 h at 37 °C triggered marked WPB degranulation (**c**,**e**,**f**). Degranulation was not observed in HUVECs treated with 10 nM 4α-PMA (**d**,**e**) (*, one-way ANOVA, *n* = 3; *p* < 0.001 compared to control). Scale bar = 20 µm.

We treated cultured HUVECs with LC *n*-3 PUFAs, DHA or EPA and examined their ability to attenuate PMA-stimulated WPB degranulation. Immunoreactive staining for vWF was observed in HUVECs, and this was localized to rod-shaped WPBs within the cytoplasm (Figure 1b). Upon stimulation of the cells with PMA, almost all cells (~97%) underwent degranulation, as evidenced by a loss of granular immunoreactive staining (Figure 1c,e; paired *t*-test, *p* < 0.05, *n* = 3). Degranulation was not observed when cells were exposed to the inactive PMA analogue, 4α-PMA (Figure 1d,e). Degranulation of WPBs was time- and concentration dependent, consistent with previous findings by Fiedler *et al.* [6]. In our study, the maximal effect was evident after 6 h incubation with 10 nM PMA (Figure 1d,e; one-way ANOVA, *p* < 0.001, *n* = 3).

### 2.2. Effect of Long Chain Omega-3 Fatty Acids on the Pattern of Weibel-Palade Body Degranulation

**Figure 2:**
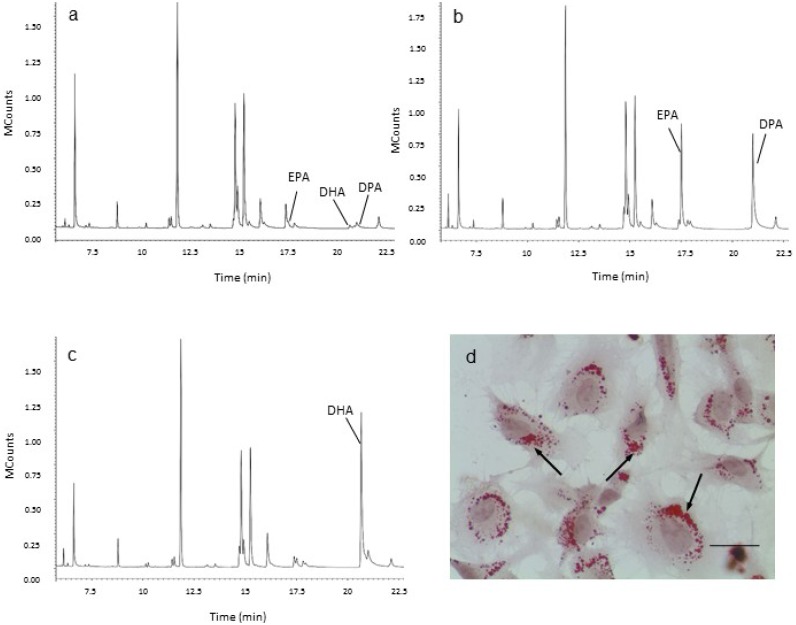
Gas Chromatography (GC) traces of human umbilical vein endothelial cells (HUVECs) treated with 120 μM eicosapentaenoic acid (EPA) or 120 μM docosahexaenoic acid (DHA) for 5 days, and lipid staining in HUVECs using Oil Red O. Basal levels of EPA and DHA, determined using GC, were low in untreated cells (**a**). Increased concentrations of EPA and DPA were detected in cells treated with EPA (**b**). An increased concentration of DHA was detected in cells treated with DHA (**c**). Oil Red O staining was negligible in untreated cells (not shown), with intense staining detected in the perinuclear region of cells that were treated with the LC *n*-3 PUFAs (**d**, arrows indicate staining in DHA treated cells). Scale bar = 25 µm.

**Figure 3:**
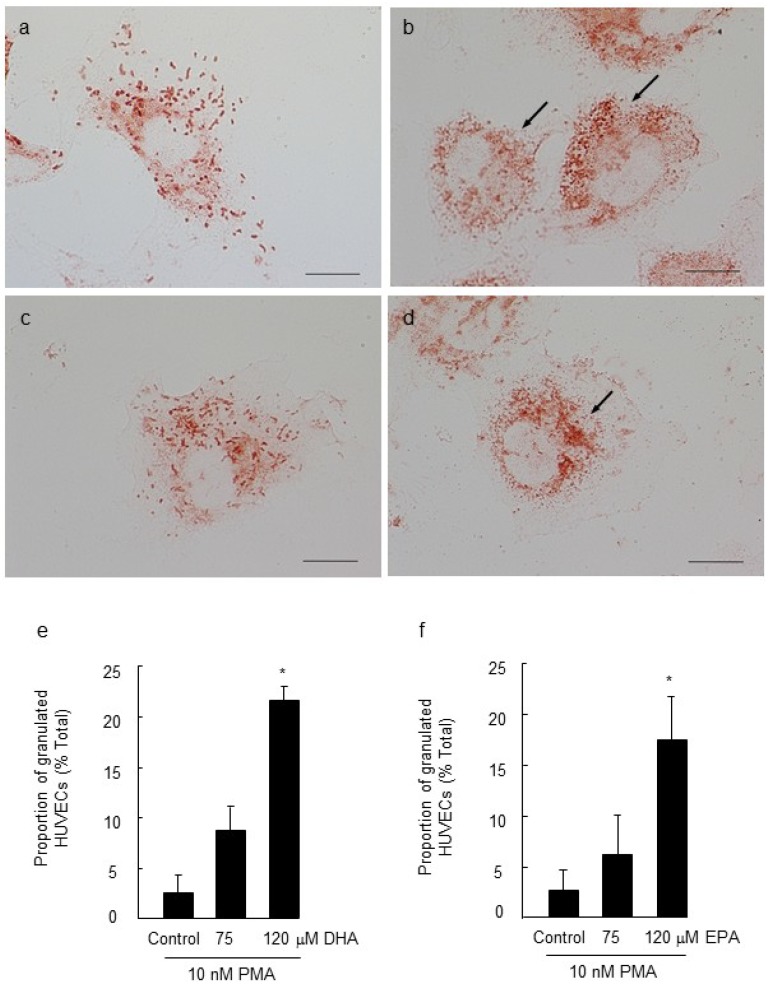
Effect of 5-day pre-treatment of human umbilical vein endothelial cells (HUVECs) with 75 μM or 120 μM docosahexaenoic acid (DHA) or 75 μM or 120 μM eicosapentaenoic acid (EPA) on Weibel-Palade body (WPB) degranulation in cells exposed to 10 nM PMA (6 h, 37 °C). Exposure of cells to DHA alone (**a**), or EPA alone (**c**) did not affect the pattern of von Willebrand factor staining. An increase in proportion of cells containing von Willebrand factor-positive granules was observed in cells treated with 120 μM DHA (**e**) or 120 μM EPA (**f**) prior to exposure of cells to PMA (*, paired *t*-test, *n* = 4; *p* < 0.05). Granules were rounded and localized to the perinuclear region (arrows; **b**,**d**). Scale bar = 20 µm.

Following 5-day incubation of HUVECs with 120 μM DHA or EPA, cellular content of DHA and EPA was increased when compared to cells incubated with media alone, as shown by GC analysis (Figure 2a–c). Cells treated with EPA also showed increased levels of docosapentaenoic acid (DPA), indicating some conversion of EPA to DPA (Figure 2b; [27]). The identity of the fatty acids was confirmed using MS analysis (data not shown). The intracellular localization of Oil Red O-stained lipid droplets (Figure 2d) provided supportive evidence for the sequestration of LC *n*-3 PUFAs by the HUVECs, and is consistent with esterification of LC *n*-3 PUFAs to cholesteryl esters and triglycerides [28]. Five-day treatment with 120 μM DHA or EPA alone had no effect on the proportion of cells staining positively for vWF (media alone, 85.9% ± 2.9%; 120 μM DHA, 83.3% ± 3.3%; 120 μM EPA, 77.8% ± 7.5%), or on WPB morphology (Figure 3a,c) . However, a greater number of cells stained positively for vWF when pre-treated with DHA or EPA prior to stimulation with PMA, compared to cells that were incubated with PMA alone (Figure 3a,c; paired *t*-test, *p* < 0.05, *n* = 4). The concentrations of LC *n*-3 PUFAs used in this study (75 and 120 μM) were within the physiological plasma concentration range for DHA (110–192 μM) and EPA (56–225 μM) in healthy individuals [29]. Interestingly, the *n*-6 PUFA, arachidonic acid (AA) attenuated WPB degranulation to a similar level to that observed for EPA and DHA, whereas shorter-chain fatty acids, oleic acid (C18:1*n*-9) and linoleic acid (C18:2*n*-6) were not different to PMA-stimulated cells (data not shown). It is not known why the pro-inflammatory *n*-6 PUFA (AA) produces a similar protective effect as the anti-inflammatory *n*-3 PUFAs, EPA and DHA. One possibility is that AA, DHA and EPA are converted to lipoxin A4; resolvin D1, and resolvin E1, respectively, which have common pro-resolving activity [30,31].

Pretreatment of cells with LC *n*-3 PUFAs prior to PMA stimulation lead to an association of vWF to small, rounded granules. This pattern of staining was different to the typical rod-shaped WPBs in non-stimulated cells. The rounded granules were localized to the perinuclear region (Figure 3b,d) whereas the rod-shaped granules were more diffusely distributed throughout the cytoplasm (Figure 3a,c). The rod-shape of WPBs in unstimulated cells is attributed to an arrangement of mature vWF multimers with a pro-peptide, and production of the small spherical granules is a sign that this configuration has been disrupted [3]. The question arises as to why these small granules form in stimulated cells that have been treated with LC *n*-3 PUFAs. One possibility is that chronic exposure of cells with LC *n*-3 PUFAs, in combination with PMA stimulation, alters the packaging of vWF in the WPBs by altering the internal pH within the granules. Michaux *et al.* [3] showed that the tubular arrangement of WPBs was disrupted by neutralization of the acidic pH within the granules following treatment of cells with the ionophore, monensin. In that study, the granules became small and spherical and the filaments of vWF in the granules were short, with reduced capacity for platelet recruitment [3]. The secretagogue-resistant granules located in the perinuclear region share similar characteristics to newly formed WPBs that are deficient in the clathrin-associated adaptor protein complex, AP-1 [32]. Although a 2-week diet of 4% fish oil in mice did not alter expression of clathrin in colonic membranes [33], further studies are required to examine the effect of LC *n*-3 PUFAs on the integrity of WPB clathrin/ AP-1 coating in endothelial cells.

### 2.3. Effect of LC *n*-3 PUFAs on Actin Cytoskeletal Rearrangement in PMA Stimulated HUVECs

The perinuclear clustering of WPBs observed in this study suggests that LC *n*-3 PUFAs might interfere with cytoskeletal remodeling needed for complete WPB degranulation. Vischer *et al.* [13] showed that actin and myosin filaments were re-arranged into prominent stress fibers only in HUVECs that had completely degranulated in response to histamine, but not in cells that were refractory to histamine. It was concluded that histamine increased intracellular calcium concentrations to induce WPB transport from the trans-golgi network to the plasma membrane [13]. In the same study, treatment of HUVECs with forskolin was shown to increase cAMP levels and to cause degranulation of peripheral WPBs but not perinuclear WPBs [13]. Interestingly, forskolin also stimulated the formation of a thick, linear peripheral actin rim [13]. Both of these changes are consistent with the appearance of some PMA-stimulated HUVECs that were pre-treated with EPA and DHA in our study, suggesting that LC *n*-3 PUFAs might augment cAMP activity in HUVECs. There is some evidence for this latter hypothesis, where it was shown that EPA can increase the production of cAMP in colonic epithelial cells via a “store-operated” mechanism [34].

**Figure 4:**
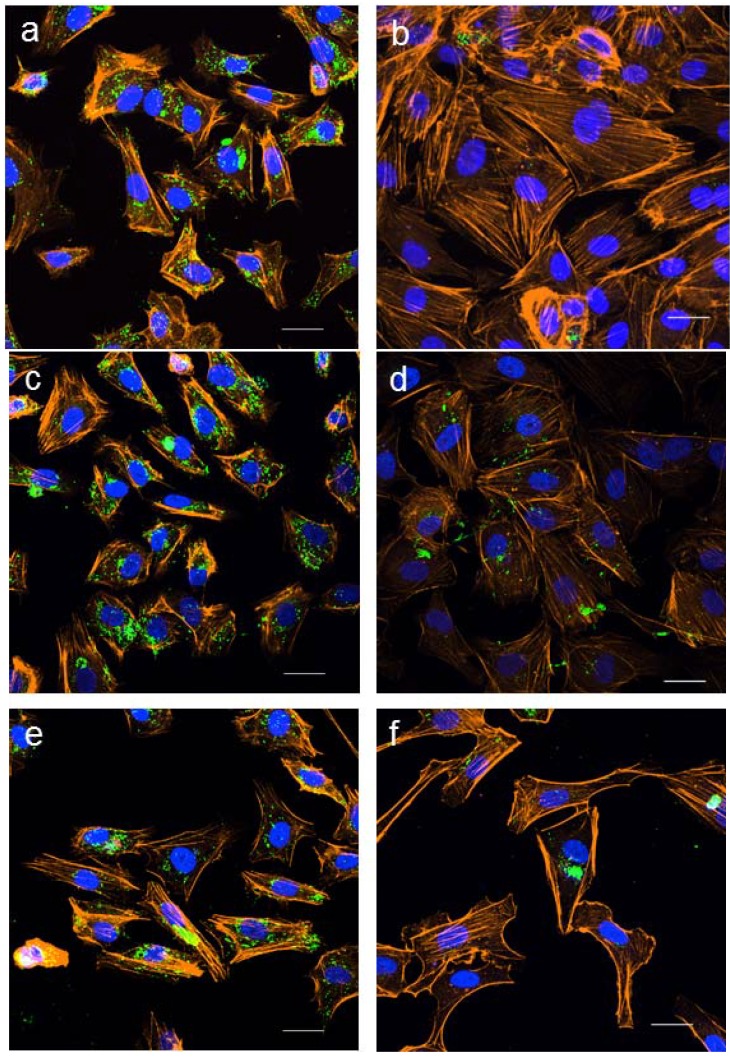
Effect of 5-day pre-treatment of human umbilical vein endothelial cells (HUVECs) with 120 μM docosahexaenoic acid (DHA) or 120 μM eicosapentaenoic acid (EPA) on actin filament rearrangement and Weibel-Palade body (WPB) degranulation. Unstimulated HUVECs stained positively for vWF with localization to WPBs (**a**). Exposure of HUVECs to PMA (10 nM, 6 h) caused the formation of prominent stress fibers throughout the cytoplasm as well as degranulation of WPBs (**b**). EPA (**c**) and DHA (**e**) alone had no effect on the diffuse localization of actin filaments and did not alter WPB distribution. Some HUVECs exposed to EPA (**d**) and DHA (**f**) prior to PMA-stimulation were protected from complete degranulation. Composite images show nuclei (blue), vWF (green) and actin (amber). Images are representative of *n* = 3 experiments. Scale bar = 25 µm.

Actin reorganization is crucial for WPB degranulation. For example, the actin stabilizing agent, jasplakinolide, inhibited both actin reorganization and proteinase activated receptor (PAR_2_) agonist-stimulated release of vWF from endothelial cells [35]. The ability of LC *n*-3 PUFAs to interfere with actin remodeling has been described previously in an *in vitro* wound-healing model [36]. Exposure of murine endothelial cells to a mixture containing 100 μM EPA and DHA ethyl esters resulted in partial disassembly of the actin cytoskeleton, which was associated with an impaired migration of the endothelial cells into a wound. We investigated whether EPA and DHA could also affect actin cytoskeletal reorganization associated with WPB degranulation in human endothelial cells. In our study, unstimulated HUVECs, and HUVECs exposed to 120 μM EPA or DHA for 5 days showed thin cortical actin rims along their margins as well as very fine actin filaments throughout the cytoplasm (Figure 4a,c,e). These cells also stained positively for vWF, which was localized to WPBs. Exposure of the cells to PMA caused rearrangement of the actin cytoskeleton into prominent stress fibers, and these were typically arranged parallel to the longitudinal axis of the cells (Figure 4b). However, some cells that were exposed to EPA or DHA prior to PMA-stimulation had an outer actin rim that was thicker and more linear, without prominent stress fiber formation across the cellular cytoplasm (Figure 4d,f), compared to cells exposed to PMA alone. Consistent with the results obtained using brightfield microscopy, vWF was localized to rounded granules in the peri-nuclear region in some cells that were exposed to EPA or DHA prior to PMA stimulation (Figure 4d,f). These findings suggest that EPA and DHA may reduce PMA-stimulated loss of perinuclear vWF by attenuating actin reorganization in the endothelial cells.

WPBs store vasoactive and pro-inflammatory mediators, and their degranulation is implicated in inflammatory disorders including hypertension and thrombosis [10,11,12]. Degranulation of WPBs is triggered by pathophysiological stimuli, including exposure of endothelial cells to mechanical stressors [37,38] and pro-inflammatory mediators such as TNF-α [39], reactive oxygen species [40], sphingolipids [41] and histamine [42]. Thus, attenuation of degranulation, for example by LC *n*-3 PUFAs, may contribute to the beneficial *in vivo* effects of LC *n*-3 PUFAs.

## 3. Experimental Section

### 3.1. Culture of Human Umbilical Vein Endothelial Cells

Umbilical cords were obtained with informed consent from women giving birth by Caesarean section at Nambour General Hospital, Queensland, Australia; with approval from the Human Research Ethics Committees of the University of the Sunshine Coast (S/09/221 & S/12/391) and the Royal Brisbane and Women’s Hospital (HREC/09/QRBW/184 & HREC/12/QRBW/99). Cords were placed in cold sterile Dulbecco’s phosphate buffered solution (PBS) and transported to the University laboratory. HUVECs were obtained using a modified method of Baudin *et al.* [43]. The umbilical vein was cannulated and flushed with Dulbecco’s PBS to remove blood. Collagenase II (1 mg/mL in M199 media) was administered into the vein, the cord was clamped at both ends and incubated for 20 min at 22 °C. The collagenase solution was retrieved from the vein, spun (400 × *g*, 5 min), and the pellet was resuspended in 20% media (M199 media containing 20% fetal calf serum, 50 μg/mL penicillin/streptomycin, 2.5 μg/mL fungizone, 2 mM glutamax-I). Cells were seeded on 25 cm^2^ collagen-coated culture flasks and grown to confluence in a humidified, 5% CO_2_ incubator at 37 °C. Cells were lifted from the flasks using 0.25% trypsin/EDTA solution and then passaged at a split ratio of 1:2, or seeded onto collagen-coated 75 cm^2^ cell culture flasks or 13 mm diameter coverslips. Media was replaced every 2 days and cells were used for assays between passage number 3 and 5.

### 3.2. Immunocytochemistry Staining for Von Willebrand Factor

Some endogenous mediators stimulate WPB degranulation through a PKC-dependent mechanism [44]. PMA was used in this study as an exogenous PKC-dependent activator of Weibel-Palade body degranulation. HUVECs were incubated in the absence or presence of PMA (1–6 h, 0.1–100 nM, 37 °C) or 4α-PMA (6 h, 10 nM), or with LC *n*-3 PUFAs (DHA or EPA at 75 or 120 μM; 5 days, *n* = 4) with or without addition of 10 nM PMA for the final 6 h. EPA (sodium salt; Nu-Check-Prep Inc., MN, USA) was dissolved in oxygen-depleted water and DHA (>99% oil; Nu-Check-Prep Inc., MN, USA) was bound to albumin as described previously [45], to produce 15 mM stock solutions. HUVECs were fixed in 4% paraformaldehyde solution for 30 min at 4 °C, washed in 0.1 M glycine (2 × 5 min, 22 °C), incubated with 3% hydrogen peroxide (5 min, 22 °C), rinsed in 0.01 M PBS (6.8 mM Na_2_HPO_4_, 2.6 mM NaH_2_PO_4_, pH 7.2), and incubated with heat-inactivated goat serum (1:20 in 0.01 M PBS, 1 h, 22 °C). Cells were incubated with a mouse monoclonal antibody to human von Willebrand factor (1:50, DAKO, clone F8/86), overnight at 22 °C in a humidified chamber. Cells were washed in 0.01 M PBS (6 × 5 min, 22 °C), incubated with anti-mouse biotin (1:200 in 0.01 M PBS; 1 h, 22 °C), washed in 0.01 M PBS (3 × 5 min, 22 °C), and then incubated with streptavidin horseradish peroxidase (HRP) (1:200 in 0.01 M PBS; 1 h, 22 °C). After a further 3 × 5 min washes in 0.01 M PBS, cells were incubated with 0.1 M acetate buffer (pH 5.3; 3 min, 22 °C), and 3-amino-9-ethylcarbazole solution for 3 min at 22 °C for detection of vWF. Cells were rinsed in distilled water. Coverslips were mounted onto microscope slides using glycerol. Photomicrographs were obtained using a Nikon DS-Fi2 camera connected to a Nikon Eclipse Ti microscope.

### 3.3. Quantitation of Weibel-Palade Body Degranulation

Cells were examined for WPB degranulation using a brightfield microscope. Cells (all cells or up to 100 cells per coverslip) were categorized as either containing vWF (granulated), or not containing vWF (degranulated), and the proportion of granulated cells to total cells was determined. Only cells with visible nuclei were included while overlapping cells were excluded.

### 3.4. Gas Chromatography—Mass Spectrometry Analysis of Cellular LC *n*-3 PUFA

HUVECs were seeded into 75 cm^2^, collagen-coated cell culture flasks and exposed to 120 μM DHA or EPA for 5 days at 37 °C. Media was removed after 5 days and a cell scraper was used to collect cells from the flasks into borosilicate test tubes. To extract phospholipids from the cells, 600 μL of methanol containing butylhydroxytoluene (BHT, 0.5 mg/mL) was added and cells were homogenized using glass rods for 1 min. Homogenized cells were covered with nitrogen gas and stored on ice for 30 min before adding 600 μL of chloroform. Cells were homogenized again for 1 min, stored on ice for 30 min and then spun (3000 × *g*, 4 °C, 5 min). Following the first spin, separation of a bottom chloroform layer and an upper methanol layer was observed. The chloroform layer, which contained the extracted lipids was withdrawn, placed in clean test tubes, covered with nitrogen gas and stored on ice until further addition of extracted lipids. The process was repeated twice, using reduced volumes of methanol with BHT and chloroform (300 μL), as well as reduced storage times on ice (10 min). During subsequent spins, the entire supernatant was withdrawn. To complete the extraction, 800 μL of chloroform and 460 μL of 0.05 M KCl was added to 1000 μL of the pooled lipid solution, mixed by vortex and spun (3000 × g, 4 °C, 10 min). The supernatant was discarded; the lipid fraction was transferred into screw top vials and dried under nitrogen gas. To hydrolyze the extracted lipids 500 μL of 9 M HCl:H_2_O:acetonitrile (1:1:18) solution containing BHT (25 mg/50 mL) was added, samples were covered with nitrogen gas and incubated overnight at 65 °C. The hydrolyzed samples were dried under nitrogen gas and freeze dried for at least 15 min before adding 250 μL of hexane and 10 μL of derivatising agent (1-*tert*-butyldimethylsilylimidazole). Samples were covered with nitrogen gas, incubated at 37 °C for 2 h and analyzed using Gas Chromatography (Varian, model 3900, Middelburg, The Netherlands) and Mass Spectrometry (Varian, model Saturn 2100T, Walnut Creek, CA, USA).

### 3.5. Oil Red O Staining for Lipids

The uptake of LC *n*-3 PUFAs acids by HUVECs was also examined using Oil Red O staining. HUVECs were seeded onto coverslips and exposed to 120 μM DHA or EPA for 5 days at 37 °C. Cells were fixed in 3.7% formaldehyde (15 min, 22 °C), stained with 0.22 μm filtered Oil Red O solution (90 min, 22 °C), and washed in Dulbecco’s PBS. The cells were counterstained with hematoxylin (5 min, 22 °C), incubated with Scott’s tap water (1 min), washed with Dulbecco’s PBS and mounted onto glass microscope slides using glycerol. Photomicrographs were obtained as described above.

### 3.6. Cellular Actin Remodeling

HUVECs were incubated in the presence of LC *n*-3 PUFAs (DHA or EPA at 120 μM; 5 days, *n* = 3) with or without addition of 10 nM PMA for the final 6h. HUVECs were fixed in 3.7% formaldehyde solution for 15 min at 22 °C, washed extensively with 1 × PBS (10 mM Na_2_HPO_4_, 1 mM KH_2_PO_4_, 140 mM NaCl, 2.6 mM KCl, pH 7.4; 3 × 5 min, 22 °C) and incubated with heat-inactivated goat serum (5% in 1 × PBS with 0.3% Triton X-100, 1 h, 22 °C). Cells were then incubated with a mouse monoclonal antibody to human von Willebrand factor (1:200 in antibody diluting buffer (1 × PBS, 1% BSA, 0.3% Triton X-100, DAKO, clone F8/86), overnight at 4 °C in a humidified chamber. Cells were washed in 1 × PBS (3 × 5 min, 22 °C) and incubated with goat anti-mouse fluorochrome-conjugated secondary antibody (1:2000 in antibody diluting buffer, Cell Signaling, IgG Fab2 Alexa-Fluor (R) 488) as well as the fluorescent nuclear stain DAPI (2 μg/mL in antibody diluting buffer, 1.5 h, 22 °C in the dark). HUVECs were washed (3 × 5 min, 22 °C), and then incubated with phalloidin-TRITC (5 μg/mL in antibody diluting buffer, Sigma Aldrich, Sydney, NSW, Australia, 40 min, 22 °C in the dark). After a further 5 × 5 min washes, coverslips were mounted onto microscope slides using glycerol. Photomicrographs were obtained using a Nikon Eclipse T*i* inverted confocal microscope. Cells were scanned using the z-stack function to obtain composite images of fluorescent staining throughout the entire thickness of the cultured HUVECs.

### 3.7. Data Analysis

Mean values were compared using paired *t*-tests or one-way ANOVA with Tukey post hoc analysis, using SPSS Statistics (IBM, Version 19, St. Leonards, NSW, Australia).

## 4. Conclusions

This study showed that chronic pre-treatment of endothelial cells with LC *n*-3 PUFAs prior to their activation lead to uptake of the fatty acids by the cells, and prevented PMA-induced stress fiber formation in some cells. These cells showed an altered pattern of endothelial exocytosis, with retention of small spherical WPBs in the perinuclear region. Since WPBs contain vasoactive and pro-inflammatory mediators, we suggest that the LC *n*-3 PUFA-dependent retention of WPB content in the presence of endothelial activators may contribute to some of their anti-inflammatory and vasoprotective effects.

## References

[1] Naya M., Tsukamoto T., Morita K., Katoh C., Furumoto T., Fujii S., Tamaki N., Tsutsui H. (2007). Plasma interleukin-6 and tumor necrosis factor-alpha can predict coronary endothelial dysfunction in hypertensive patients. Hypertens. Res..

[2] Russell F.D., Skepper J.N., Davenport A.P. (1998). Evidence using immunoelectron microscopy for regulated and constitutive pathways in the transport and release of endothelin. J. Cardiovasc. Pharmacol..

[3] Michaux G., Abbitt K.B., Collinson L.M., Haberichter S.L., Norman K.E., Cutler D.F. (2006). The physiological function of von Willebrand’s factor depends on its tubular storage in endothelial Weibel-Palade bodies. Dev. Cell.

[4] Nightingale T.D., Pattni K., Hume A.N., Seabra M.C., Cutler D.F. (2009). Rab27a and MyRIP regulate the amount and multimeric state of VWF released from endothelial cells. Blood.

[5] Metcalf D., Nightingale T., Zenner H., Lui-Roberts W., Cutler D. (2008). Formation and function of Weibel-Palade bodies. J. Cell Sci..

[6] Fiedler U., Scharpfenecker M., Koidl S., Hegen A., Grunow V., Schmidt J.M., Kriz W., Thurston G., Augustin H.G. (2004). The Tie-2 ligand angiopoietin-2 is stored in and rapidly released upon stimulation from endothelial cell Weibel-Palade bodies. Blood.

[7] Lowenstein C.J., Morrell C.N., Yamakuchi M. (2005). Regulation of Weibel-Palade body exocytosis. Trends Cardiovasc. Med..

[8] Cleator J.H., Qin Zhu W., Vaughan D.E., Hamm H.E. (2006). Differential regulation of endothelial exocytosis of P-selectin and von Willebrand factor by protease-activated receptors and cAMP. Blood.

[9] Sen U., Tyagi N., Patibandla P.K., Dean W.L., Tyagi S.C., Roberts A.M., Lominadze D. (2009). Fibrinogen-induced endothelin-1 production from endothelial cells. Am. J. Physiol. Cell Physiol..

[10] Okruhlicová L’., Dlugošová K., Mitašiková M., Bernátová I. (2008). Ultrastructural characteristics of aortic endothelial cells in borderline hypertensive rats exposed to chronic social stress. Physiol. Res..

[11] Davis J.S., Yeo T.W., Piera K.A., Woodberry T., Celermajer D.S., Stephens D.P., Anstey N.M. (2010). Angiopoietin-2 is increased in sepsis and inversely associated with nitric oxide-dependent microvascular reactivity. Crit. Care.

[12] Yeo T.W., Lampah D.A., Tjitra E., Piera K., Gitawati R., Kenangalem E., Price R.N., Anstey N.M. (2010). Greater endothelial activation, Weibel-Palade body release and host inflammatory response to *Plasmodium vivax*, compared with *Plasmodium falciparum*: A prospective study in Papua, Indonesia. J. Infect. Dis..

[13] Vischer U., Barth H., Wollheim C. (2000). Regulated von Willebrand factor secretion is associated with agonist-specific patterns of cytoskeletal remodelling in cultured endothelial cells. Arterioscler. Thromb. Vasc. Biol..

[14] Nightingale T., White I., Doyle E., Turmaine M., Harrison-Lavoie K., Webb K., Cramer L.P., Cutler D.F. (2011). Actomyosin II contractility expels von Willebrand factor from Weibel-Palade bodies during exocytosis. J. Cell Biol..

[15] Geleijnse J.M., Giltay E.J., Grobbee D.E., Donders A.R.T., Kok F.J. (2002). Blood pressure response to fish oil supplementation: Metaregression analysis of randomized trials. J. Hypertens..

[16] GISSI-Prevenzione Investigators (1999). Dietary supplementation with *n*-3 polyunsaturated fatty acids and vitamin E after myocardial infarction: Results of the GISSI-Prevenzione trial. Lancet.

[17] Bønaa K., Bjerve K., Straume B., Gram I., Thelle D. (1990). Effect of eicosapentaenoic and docosahexaenoic acids on blood pressure in hypertension—A population-based intervention trial from the Tromsø Study. N. Engl. J. Med..

[18] Mori T., Bao D., Burke V., Puddey I., Beilin L. (1999). Docosahexaenoic acid but not eicosapentaenoic acid lowers ambulatory blood pressure and heart rate in humans. Hypertension.

[19] Morris M., Sacks F., Rosner B. (1993). Does fish oil lower blood pressure? A meta-analysis of controlled trials. Circulation.

[20] De Caterina R., Cybulsky M., Clinton S., Gimbrone M., Libby P. (1994). The omega-3 fatty acid docosahexaenoate reduces cytokine-induced expression of proatherogenic and proinflammatory proteins in human endothelial cells. Arterioscler. Thromb. Vasc. Biol..

[21] Stulnig T.M., Huber J., Leitinger N., Imre E.M., Angelisova P., Nowotny P., Waldhausl W. (2001). Polyunsaturated eicosapentaenoic acid displaces proteins from membrane rafts by altering raft lipid composition. J. Biol. Chem..

[22] Webb Y., Hermida-Matsumoto L., Resh M.D. (2000). Inhibition of protein palmitoylation, raft localization, and T cell signaling by 2-bromopalmitate and polyunsaturated fatty acids. J. Biol. Chem..

[23] He K., Liu K., Daviglus M.L., Jenny N.S., Mayer-Davis E., Jiang R., Steffen L., Siscovick D., Tsai M., Herrington D. (2009). Associations of dietary long-chain *n*-3 polyunsaturated fatty acids and fish with biomarkers of inflammation and endothelial activation (from the Multi-Ethnic Study of Atherosclerosis [MESA]). Am. J. Cardiol..

[24] Ye S., Tan L., Ma J., Shi Q., Li J. (2010). Polyunsaturated docosahexaenoic acid suppresses oxidative stress induced endothelial cell calcium influx by altering lipid composition in membrane caveolar rafts. Prostaglandins Leukot. Essent. Fatty Acids.

[25] Johansen O., Seljeflot I., Høstmark A.T., Arnesen H. (1999). The Effect of supplementation with omega-3 fatty acids on soluble markers of endothelial function in patients with coronary heart disease. Arterioscler. Thromb. Vasc. Biol..

[26] Seljeflot I., Arnesen H., Brude I.R., Nenseter M.S., Drevon C.A., Hjermann I. (1998). Effects of omega-3 farry acids and/or antioxidants on endothelial cell markers. Eur. J. Clin. Invest..

[27] Kanayasu-Toyoda T., Morita I., Murota S. (1996). Docosapentaenoic acid (22:5, *n*-3), an elongation metabolite of eicosapentaenoic acid (20:5, *n*-3), is a potent stimulator of endothelial cell migration on pretreatment *in vitro*. Prostaglandins Leukot. Essent. Fatty Acids.

[28] McIntosh A.L., Huang H., Atshaves B.P., Wellberg E., Kuklev D.V., Smith W.L., Kier A.B., Schroeder F. (2010). Fluorescent *n*-3 and *n*-6 very long chain polyunsaturated fatty acids: Three-photon imaging in living cells expressing liver fatty acid-binding protein. J. Biol. Chem..

[29] Salm P., Taylor P.J., Kostner K. (2011). Simultaneous quantification of total eicosapentaenoic acid, docosahexaenoic acid and arachidonic acid in plasma by high-performance liquid chromatography-tandem mass spectrometry. Biomed. Chromatogr..

[30] Levy B.D., Clish C.B., Schmidt B., Gronert K., Serhan C.N. (2001). Lipid mediator class switching during acute inflammation: signals in resolution. Nat. Immunol..

[31] Weylandt K.H., Chiu C.-Y., Gomolka B., Waechter S.F. (2012). Omega-3 fatty acids and their lipid mediators: Towards an understanding of resolvin and protectin formation: omega-3 fatty acids and their resolvin/protectin mediators. Prostaglandins Lipid Mediat..

[32] Lui-Roberts W.W.Y., Collinson L.M., Hewlett L.J., Michaux G., Cutler D.F. (2005). An AP-1/clathrin coat plays a novel and essential role in forming the Weibel-Palade bodies of endothelial cells. J. Cell Biol..

[33] Ma D.W., Seo J., Davidson L.A., Callaway E.S., Fan Y.Y., Lupton J.R., Chapkin R.S. (2004). *N*-3 PUFA alter caveolae lipid composition and resident protein localization in mouse colon. FASEB J..

[34] Roy J., Lefkimmiatis K., Moyer M.P., Curci S., Hofer A.M. (2010). The ω-3 fatty acid eicosapentaenoic acid elicits cAMP generation in colonic epithelial cells via “store-operated” mechanism. Am. J. Physiol. Gastrointest. Liver Physiol..

[35] Klarenbach S., Chipiuk A., Nelson R., Hollenberg M., Murray A. (2003). Differential actions of PAR2 and PAR1 in stimulating human endothelial cell exocytosis and permeability: The role of Rho-GTPases. Circ. Res..

[36] Tonutti L., Manzi L., Tacconi M.T., Bazzoni G. (2010). Eicosapentaenoic acid inhibits endothelial cell migration *in vitro*. J. Angiogenes. Res..

[37] Macarthur H., Warner T., Wood E., Corder R., Vane J. (1994). Endothelin-1 release from endothelial cells in culture is elevated both acutely and chronically by short periods of mechanical stretch. Biochem. Biophys. Res. Commun..

[38] Galbusera M., Zoja C., Donadelli R., Paris S., Morigi M., Benigni A., Figliuzzi M., Remuzzi G., Remuzzi A. (1997). Fluid shear stress modulates von Willebrand Factor release from human vascular endothelium. Blood.

[39] Kaye S.A., Obrig T.G. (1995). Effect of TNF-α, shiga toxin and calcium ionophore on Weibel-Palade body content of endothelial cells: possible implications for the hemolytic uremic syndrome. Thromb. Res..

[40] Takanaro M., Meneshian A., Sheikh E., Yamakawa Y., Wilkins K.B., Hopkins E.A., Bulkley G.B. (2002). Rapid upregulation of endothelial P-selectin expression via reactive oxygen species generation. Am. J. Physiol. Heart Circ. Physiol..

[41] Bhatia R., Matsushita K., Yamakuchi M., Morrell C.N., Cao W., Lowenstein C.J. (2004). Ceramide triggers Weibel-Palade body exocytosis. Circ. Res..

[42] Erent M., Meli A., Moisoi N., Babich V., Hannah M., Skehel P., Knipe L., Zupancic G., Ogden D., Carter T. (2007). Rate, extent and concentration dependence of histamine-evoked Weibel-Palade body exocytosis determined from individual fusion events in human endothelial cells. J. Physiol..

[43] Baudin B., Bruneel A., Bosselut N., Vaubourdolle M. (2007). A protocol for isolation and culture of human umbilical vein endothelial cells. Nat. Protoc..

[44] Ge X., Low B., Liang M., Fu J. (2007). Angiotensin II directly triggers endothelial exocytosis via protein kinase C-dependent protein kinase D2 activation. J. Pharmacol. Sci..

[45] Tardivel S., Gousset-Dupont A., Robert V., Pourci M.-L., Grynberg A., Lacour B. (2009). Protective effects of EPA and deleterious effects of DHA on eNOS activity in Ea hy 926 cultured with lysophosphatidylcholine. Lipids.

